# Fungal biodegradation of chlorinated herbicides: an overview with an emphasis on 2,4-D in Argentina

**DOI:** 10.1007/s10532-023-10022-9

**Published:** 2023-02-25

**Authors:** Karen Magnoli, Cecilia Carranza, Melisa Aluffi, Carina Magnoli, Carla Barberis

**Affiliations:** 1 Departamento de Microbiología e Inmunología, Facultad de Ciencias Exactas, Físico-Químicas y Naturales, Instituto de Investigación en Micología y Micotoxicología (IMICO-CONICET), Universidad Nacional de Río Cuarto, Ruta Nacional Nº 36 Km 601 (5800) Río Cuarto, Córdoba, Argentina; 2 Fellowship of CONICET, Córdoba, Argentina; 3 Member of the Research Career of CONICET, Córdoba, Argentina

**Keywords:** Chlorinated herbicides, Bioremediation, Fungal species, Regulatory aspects, Characterization of 2,4-dichlorophenoxyacetic acid-removing strains

## Abstract

Chlorinated herbicides are one of the main types of pesticide used in agriculture. In Argentina, 2,4-dichlorophenoxyacetic acid (2,4-D) is the most applied herbicide for the control of broadleaf weeds, but the risks it poses for the environment and human health are cause for great concern. A promising technology to remove this kind of pollutants, or neutralize them in such a way that they become less or non-toxic, is the use of degrading or detoxifying microorganisms from contaminated sites. Filamentous fungi can bioremediate xenobiotics thanks to their efficient enzymatic machinery. However, most studies on the degradation of 2,4-D have been carried out with bacteria, and little is known about whether it can be efficiently biodegraded by fungi. In the environment, fungal strains and native microbiota may detoxify contaminants through mechanisms like biosorption, bioabsortion, biotransformation, and/or degradation. Whether these processes occur separately or simultaneously depends on the metabolic ability of the strains that conform the microbial community. Another important concern when attempting to introduce detoxifying microorganisms into a contaminated environment is the GRAS (“Generally Recognized As Safe”) assessment or status. These are studies that help predict a biodegrading microorganism’s pathogenicity, toxicity**,** and infectivity before in situ application. This application, moreover, is regulated by different legal frameworks. The present review aims to outline the main aspects of 2,4-D degradation by fungi, and to summarize the current state of research on the topic in Argentina.

## Introduction

Growing urbanization and the accompanying demand for high quality food has resulted in the implementation of strategies to improve crop yields. These strategies include direct seeding, the use of new machinery and irrigation systems, and the massive application of fertilizers and pesticides to reduce economic and nutritional losses. Forty percent of the pesticides used worldwide are organochlorine or chlorinated chemicals. They are composed of carbon, hydrogen**,** and oxygen atoms, and they have chlorine-substituted aliphatic or aromatic rings. They are non-polar and can accumulate in animal tissues, which means they are transferred into the food chain and thus pose a risk of toxicity to animals and humans alike (Jayaraj et al. 2016).

Among organochlorine pesticides, the most used in South America are herbicides based on 2,4-dichlorophenoxyacetic acid (2,4-D), a synthetic plant auxin. The World Health Organization considers it a moderately hazardous, class II herbicide (WHO 2019), and the International Agency for Research on Cancer (IARC) has classified it as possibly carcinogenic to humans (Group 2B) (IARC 2018). It is applied on rice, wheat, sorghum, sugar cane and corn to control broadleaf weeds, and the recommended doses are between 0.5 and 2 L ha^−1^ depending on the crop (WHO 2019). However, its massive use and the mismanagement of related waste and effluents produce a large number of pollutants. According to different reports, only between 0.1 and 5% of the total pesticides applied actually get to the target pests, while the rest ends up in the soil and water (Nawaz et al. 2011). More specifically, hydrophobic herbicides like chlorinated compounds are often adsorbed and retained within soil particles and organic matter, whereas water-soluble herbicides enter surface and groundwater bodies through percolation, runoff**,** and drainage. Some characteristics of 2,4-D, like its high molecular mass, halogenation, tendency towards bioaccumulation, and lipophilicity, make it more recalcitrant or persistent and thus increase its chances of contaminating air, water**,** and soil fractions when it is used at higher concentrations than those recommended (Kennepohl et al. 2010).

There are different options for the remediation and cleanup of these compounds. Techniques such as chemical precipitation, oxidation–reduction, filtration, ion exchange, and electrochemical treatment can remove them from the soil, water**,** and air, but are not sufficiently effective (Ortiz Hernández et al. 2013; Agarry et al. 2020). Although the reaction times behind these processes are very fast, they may lead to incomplete degradation. In addition, they are costly and require specific instruments, materials, and hazardous chemicals (Bhadouria et al. 2020). A promising alternative is the use of microorganisms with the ability to degrade or render less harmful a wide range of toxic xenobiotics, i.e.**,** bioremediation, which is much more environmentally friendly and cost-effective than the physical/chemical methods (Maqbool et al. 2016). Bioremediation of chlorinated agrochemicals has been extensively studied using bacteria, since they are easy to manipulate and grow rapidly, but some fungal strains that can detoxify and degrade these chemicals have also been isolated and characterized (Serbent et al. 2019). In the specific case of Argentina, despite growing concerns about 2,4-D moderate toxicity, no fungal strains which may be able to stimulate its degradation have so far been characterized or isolated from contaminated sites (Corcoran et al. 2020). Similarly, very little is known about the enzymes and pathways involved in the fungal degradation of 2,4-D herbicides (Vroumsia 2005). The present review outlines the mechanisms through which fungi remove and degrade chlorinated herbicides, mainly those based on 2,4-D. It also aims to emphasize the importance of further exploring these mechanisms for the development of successful bioremediation schemes. Finally, it considers some aspects of 2,4-D use in Argentina and the legal framework that regulates it.

## Fungi as degraders of chlorinated herbicides

The indiscriminate use and poor management of herbicides affect human and animal health, as well as the environment. Chlorinated herbicides are especially persistent (Kaur et al. 2008) due to the presence of chlorine-substituted, polar functional groups in their molecules, their cyclic structure, and their lipophilic nature (Bose et al. 2021). These characteristics allow them to become accumulated in human and animal tissue and the environment (Briz et al. 2011; Chaussonnerie et al. 2016). Table 1 shows the most widely used organochlorine herbicides worldwide, their chemical structure, and their degree of persistence in the environment.

**Table 1:** Main chlorinated herbicides used in agriculture worldwide

Chemical name	Chemical structure	Use	Toxicity classification (WHO) and LD50 (mg kg^−1^)	Persistence in environment	CAS Nº
2,4-dichlorophenoxyacetic acid (2,4-D)		Herbicide (agricultural and garden application)	Moderately hazardous (375)	7–40 days	94–75–7
4-(2,4-dichlorophenoxy) butyric acid (2,4-DB)		Herbicide (agricultural and garden application)	Moderately hazardous (700)	Data not shown	94–82–6
2-methyl-4-chlorophenoxyacetic acid (MCPA)		Herbicide (agricultural and garden application)	Moderately hazardous (700)	41 days	94–74–6
2,4,5-Trichloropyrimidine (2,4,5-T)		Herbicide (agricultural application—totally prohibited)	Highly hazardous (500)	Data not show	93–76–5
2-(2,4,-trichlorophenoxy) propanoic acid (Fenoprop, Silvex)		Herbicide (agricultural application—totally prohibited)	Highly hazardous (650)	Data not show	93–72–1
Dicamba		Herbicide (agricultural application)	Moderately hazardous (1707)	31 days	1918–00–9

*WHO* World health organization. *DL50* acute lethal doses 50, amount of a substance that can be expected to cause death in 50% of the rats. *CAS* chemical abstracts service registry number. *Source* Bokade et al. 2021

Fungi are a promising alternative to address this problem. They are agents of biological recycling that have evolved an extensive range of enzymes, metabolic pathways, and control mechanisms to degrade pesticides. (Maqbool et al. 2016). Their biotechnological application has several benefits. Fungal mycelia can penetrate a great diversity of substrates, and they grow robustly and massively on small surfaces. Their populations are genetically stable and adapt well to physical and chemical fluctuations related to pH, temperature, redox potential, and the presence of xenobiotics. Moreover, they have evolved an extensive range of enzymes, metabolic pathways, and control mechanisms to degrade pesticides (Maqbool et al. 2016). Unlike that of bacteria, their enzymatic machinery has broad subject specificity. It includes extracellular and intracellular enzymes like peroxidases (manganese and lignin peroxidase), laccases, esterases, transferases, and cytochrome P450 monooxygenases, dioxygenases, hydrolases, and dehalogenases. These enzymes catalyze the oxidation of a variety of chlorinated herbicides and thus play a significant role in their degradation (Maqbool et al. 2016; Deshmukh et al. 2016; Bose et al. 2021; Bokade et al. 2021). Table 2 summarizes the fungal species which have recently been reported as degraders of chlorinated pesticides.

**Table 2:** Fungal species reported as degraders of chlorinated herbicides in recent years

Fungi	Chlorinated pesticide	Concentration degraded	Degradation percentage and time of incubation	References
*Trametes versicolor*	Atrazine	40 mg kg^−1^	45% during 60 days	Lizano-Fallas et al. (2017)
	Chlorpyrifos		65% during 20 days	
*Anthracophyllum discolor* SP4 CCCT 16.5	Atrazine	60 mg kg^−1^	96% during 30 days	Elgueta et al. (2016)
*Abortiporus biennis*	Chlorpyrifos	200 mg L^−1^	79% during 17 days	Rivero et al. (2016)
*A.discolor*	Atrazine	35 mg L^−1^	> 90% during 30 days	Diez et al. (2015)
	Chlorpiryfos			
*Trichoderma koningii*	Alachlor	50 mg L^−1^	90% during 72 h	Nykiel-Szymańska et al. (2018b)
*Coriolus versicolor, Hypholoma fasciculare *and* Stereum hirsutum*	Atrazine, diuron and chlorpyrifos	10 mg L^-1^	> 86% during 42 days	Bending et al. (2002)

**Fig. 1:**
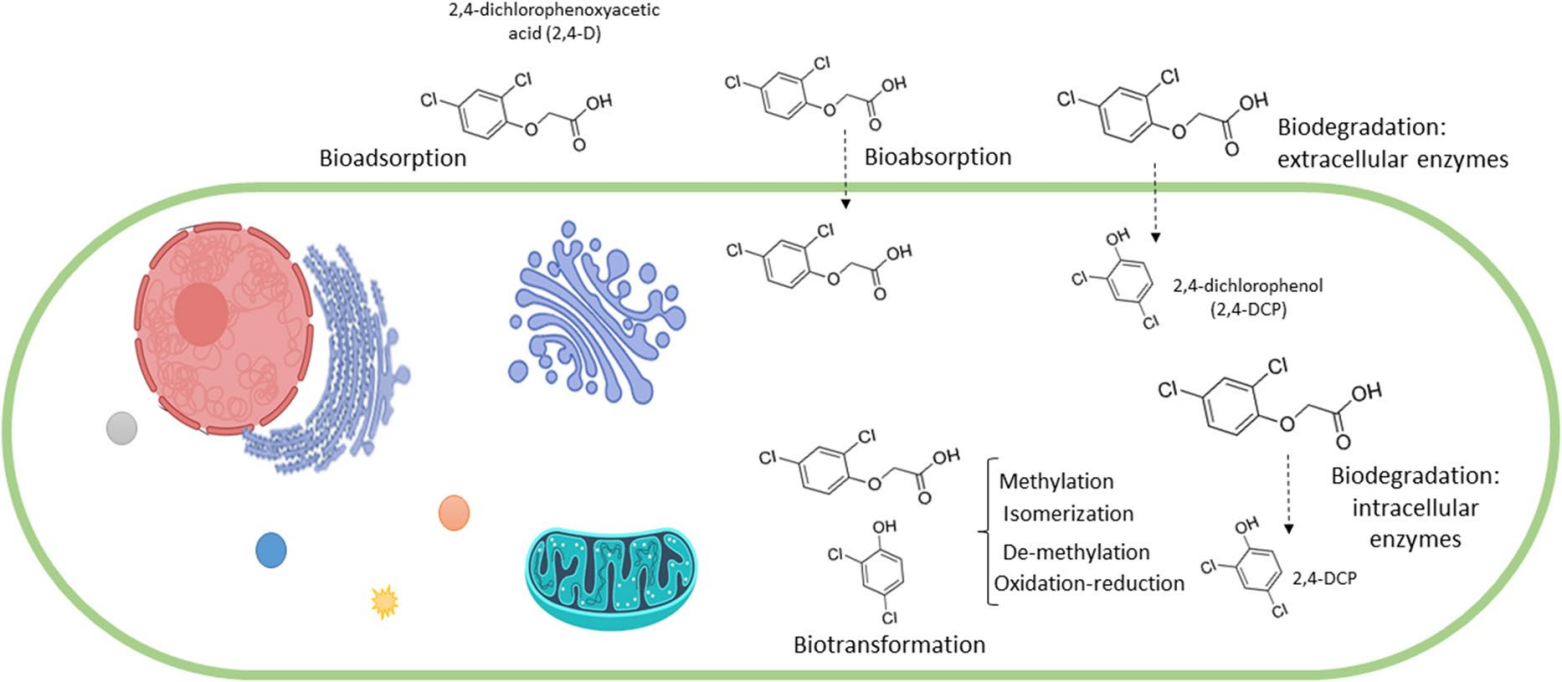
Mechanisms involved in fungal bioremediation of herbicides

Some studies have described the ability of fungal strains to remove 2,4-D herbicides from different matrices (Vroumsia et al. 2005; Ferreira-Guedes et al. 2012; Bhosle and Thore 2016; Nykiel-Szymańska et al. 2018a). However, not enough is known about the genes and enzymes involved. Figure 1 shows mechanisms through which fungi can bioremediate herbicides. Some of them are non-enzymatic, like bioaccumulation, while others are enzymatic, like biotransformation and biodegradation (Bokade et al. 2021).

Bioaccumulation is the sequestration of chemicals through the synthesis of intracellular chelates and their posterior dilution inside fungal cells. This process may involve transporting the contaminants from the internal fungal cell biomass to internal organelles (Deshmukh et al. 2016; Bokade et al. 2021). Less frequently, it may also occur when a minor structural change renders the parent molecule less or non-toxic (e.g., methylation, demethylation, oxidation, dehalogenation, hydroxylation, and reduction) (Olicón-Hernández et al. 2017; Singh et al. 2019; Kumar et al. 2021). In general, however, contaminants become bioaccumulated either through biosorption or bioabsorption (see next section).

Biotransformation and biodegradation are the main mechanisms used by fungi for detoxifying or removing herbicides (Singh 2017). The compounds released into the environment as a result of biotransformation have a different molecular structure, which often increases their solubility and decreases their biological activity, making them susceptible to degradation by other microorganisms that share the same habitat as the fungi (Bertrand et al. 2015).

Fungi may degrade herbicides to satisfy different nutritional needs. For example, they can use them as a source of carbon and energy when there are no other sources available. Certain enzymes are synthesized for this purpose, and the result is the complete mineralization of the chemical. This mode of degradation, called metabolism, is functionally like what happens when natural nutrients are degraded and consumed. Co-metabolism, on the other hand, is what takes place when the fungal species can degrade the herbicide without using it as a source of carbon and energy. In other words, some fungal strains can degrade a wide range of herbicides without directly engaging in the metabolic process (Bhadouria et al. 2020). They do this by synthetizing biosurfactants (sophorolipids, glycolipoproteins, glycolipids), active compounds with hydrophilic and hydrophobic portions that interact with differently polarized phases. This leads to the dispersion of organic chemicals into small droplets, which reduces their surface tension and increases their bioavailability for fungi (Bokade et al. 2021). Such compounds are considered an important bioremediation tool (Olicón-Hernández et al. 2017).

## Mechanisms and pathways involved in the fungal bioremediation of chlorinated herbicides

As mentioned before, fungi are able to remove herbicides from the environment through different mechanisms (Deshmukh et al. 2016; Olicón-Hernández et al. 2017; Kumar et al. 2021). For any of these to be effective, the first step is to make the contaminant more bioavailable to the fungus, i.e., to facilitate the interaction between the two so that the toxic molecules may enter the fungal cell more easily. This is accomplished by subjecting the contaminant’s structure to physical and chemical changes that render it less mobile and thus more available to the living system, in a process called immobilization (Deshmukh et al. 2016). The functional groups in the fungal cell wall are crucial at this stage. After being immobilized, the contaminant may undergo biosorption/bioabsorption, biotransformation, and/or biodegradation.

Biosorption is the adsorption of the chemical compounds on the surface of the mycelium through chelation, precipitation, reduction, and/or ion exchange. Fungal biomass is a useful biosorbent due to the presence of polymeric biomaterials such as glucans, chitin, and glycoproteins in the fungal wall. These components not only help fungi to maintain the shape, strength, and integrity of the cell structure (Viraraghavan and Srinivasan 2011); they also facilitate the initial interaction with the contaminant (Aksu 2005; Kumar et al. 2021).

Bioremediation based on biosorption is highly selective, efficient, and cost effective, and can be carried out with living or dead fungal biomass. When using living biomass, its viability must be maintained during adsorption through a continuous supply of nutrients. Dead biomass is more advantageous for removing toxic compounds from water, since it does not require a constant nutrient source and can be regenerated and reused throughout many cycles (Aksu 2005). In recent years, certain fungal species have been specifically studied for their ability to adsorb and remove chloro-phenols and chlorinated herbicides (Bayramoglu et al. 2009; Viraraghavan and Srinivasan 2011; Legorreta-Castañeda et al. 2020). According to some of these reports, chloro-phenols are more effectively adsorbed than phenols (Bayramoglu et al. 2009). This is because their chlorine substituent can activate the aromatic ring, which favors donor–acceptor interactions between their phenolic component and the groups on the biosorbent surface (Wu and Yu 2006). Biosorption capacity is also affected by the position of the chlorine group on the ring. In the *para* position, the group is biosorbed better than in the *ortho* position. This could be explained by a steric hindrance between –Cl and –OH in the case of *ortho*-chorophenol (Aksu 2005).

On the other hand, bioabsorption consists of the incorporation of contaminants into the fungal mass. It depends on fungi producing extracellular enzymes which convert complex chemical compounds into simpler ones (Gadd 2001), so that they may be uptaken by the fungal cell through active transportation or passive diffusion. Once inside the cell, the bioavailability of the compounds decreases and they might become less toxic after undergoing biotransformation, biodegradation, sequestration or biochelation (Bokade et al. 2021). These processes can be harnessed for the removal of herbicides, insecticides, or polycyclic aromatic hydrocarbons (PAHs), among others (Ortiz Hernández et al. 2013).

Biotransformation occurs when the molecular structure of the chemical is modified to reduce its toxicity or make it non-toxic for the cell (Singh 2017; Bokade et al. 2021). These modifications can take the form of oxidations, reductions, hydrolysis, isomerization, or the introduction of new carbon structures (Parkinson and Ogilvie 2008). Furthermore, they may occur inside living cells or be catalyzed outside the cell by extracellular enzymes or enzymes from lysed cells (Hüttel and Hoffmeister 2011). For example, the basidiomycete *Irpex consors* converted 70% of 0.5 g of chlorinated herbicide dimethenamid-P (DMTA-P) in liquid cultures within 6 days. As a result, nine DMTA-P products were identified (Imami et al. 2016).

Biodegradation, in which more than one microorganism is involved, entails the rupture of toxic organic compounds into other compounds with similar or less toxicity (Bansal 2012). Biodegrading microorganisms that belong to soil and aquatic microbiota interact with each other through the transfer of substrates and products to obtain energy and nutrients. Filamentous fungi are model producers of extracellular and intracellular enzymes and therefore play an important role in the biodegradation of xenobiotics, especially those not readily degraded by bacteria (Ortiz Hernández et al. 2013).

**Fig. 2:**
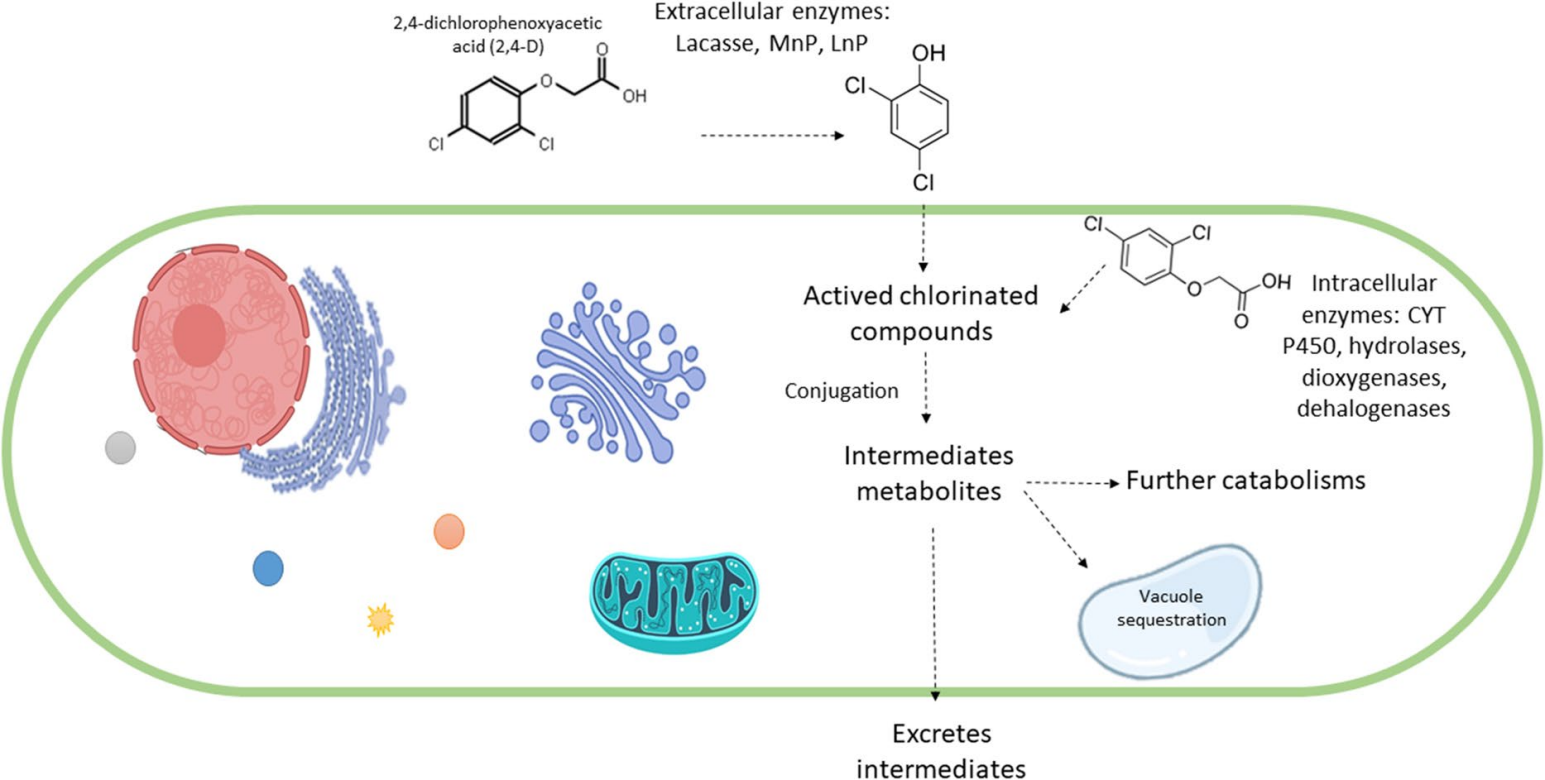
Performance of different fungal enzymes in biodegradation

The degradation of herbicides typically comprises three phases (Fig. 2), the first of which is biotransformation. As explained above, reactions such as oxidations, reductions, and/or hydrolysis produce changes in the chemical and biological properties of the parent molecule. Although in general the ensuing metabolites are less toxic and more soluble than the original compound, the presence of halogenated substituents means this is not the case for chlorophenolic herbicides (Bertrand et al. 2015). In the second phase, conjugation reactions take place where a sugar or amino acid molecule can bind to a parent herbicide compound or a degradation metabolite, with the aim of decreasing toxicity and enhancing water solubility (Devault and Karolak 2020). Finally, there is a secondary conjugation of the metabolites from the second phase, which are even less toxic than the previous ones. In the best case, these degradation metabolites integrate central metabolic pathways for the synthesis of microbial biomass (Ortiz Hernández et al. 2013). Whether one, two or all three phases occur depends on the enzymatic machinery of each fungal species (Gadd 2001). The degradation of organochlorine compounds involves enzymes that belong to different functional classes, or pathways that produce different metabolic intermediates. These metabolites compete with other organic compounds to be used by fungi as nutrient sources (Deshmukh et al. 2016).

## A brief chronology of 2,4-D-based herbicides

Chlorinated herbicides have been classified by the Stockholm Convention as dangerous compounds due to their persistence and potential for bioaccumulation. Although most of them have been banned, they are still used in many countries or have been replaced by similar formulations which have yet to be prohibited (UNEP 2019). The latter has happened in Argentina with herbicides that contain 2,4-D as the active principle, whose ester formulations have been replaced by amine formulations (SENASA 2019).

**Fig. 3:**
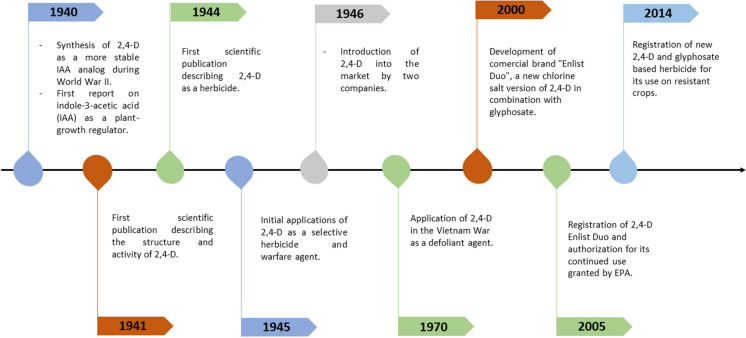
Brief history of 2,4-D herbicide

Figure 3 provides a brief historical outline of the production and use of 2,4-D. It was initially synthesized in 1940, during World War II, by four research groups working independently of each other in the United Kingdom and the United States (Hamner and Tukey 1944). The researchers were subject to wartime secrecy laws and did not follow the usual procedures for publication and patent disclosure. The same year, hormone indole-3-acetic acid (IAA), a plant auxin, was reported as a plant growth regulator when used at high concentrations. Its ability to kill broadleaf weeds on a cereal field was also described (Quastel 1950). Two phenoxy herbicides which were analogous to IAA but more stable were soon synthesized in the US: 2,4-dichlorophenoxyacetic acid (2,4-D) (Quastel 1950) and 2,4,5-trichlorophenoxyacetic acid (2,4,5-T) (Peterson 1967). In 1941, a scientific publication described for the first time the structure of 2,4-D and its plant growth-regulating activity (Pokorny 1941). In 1944, it was characterized as selective, and in 1945 it started being applied for war purposes (Hamner and Tukey 1944; Quastel 1950): both Britain and the US used it as a warfare agent against potato and rice crops in Germany and Japan (Peterson 1967; Cobb and Reade 2010).

In 1946, a year after the war ended, the firm Dow AgroSciences began commercializing 2,4-D as an herbicide to control broadleaf weeds in rice, wheat, sorghum, sugar cane, and grasslands (Peterson 1967). The same year, the American Chemical Paint Company introduced 2,4-D into the market as an herbicide called "Weedone" (Quastel 1950). In 1970, the US Army used “Agent Orange”, an herbicide and defoliant made up of equal parts of 2,4-D and 2,4,5-T, to damage the jungle where Vietnamese soldiers sought refuge. The dioxins produced by 2,4,5-T were responsible for serious health damage in people who had been exposed to the agent (Martini 2012).

In the 2000s, DowAgroSciences combined a new chlorine version of 2,4-D (2,4-D chlorine) with glyphosate under the brand “Enlist Duo'”. This new formulation was created to control broadleaf weeds that were beginning to show resistance to glyphosate (EPA 2014). A registration eligibility re-evaluation of 2,4-D was conducted by the United States Environmental Protection Agency (EPA) and Health Canada in 2005, and both agencies decided that it was eligible for continued use (USDA 2005; Dow AgroSciences 2011). In 2014, EPA registered a new herbicide with 2,4-D choline salt and glyphosate dimethyl ammonium salt as the active ingredients, to be used on corn and soybean that were genetically modified to resist both 2,4-D and glyphosate (EPA 2014). In the last decade, more than 600 products whose active principle is 2,4-D have been released (Song 2014).

## Fungi involved in the degradation of 2,4-D

**Fig. 4:**
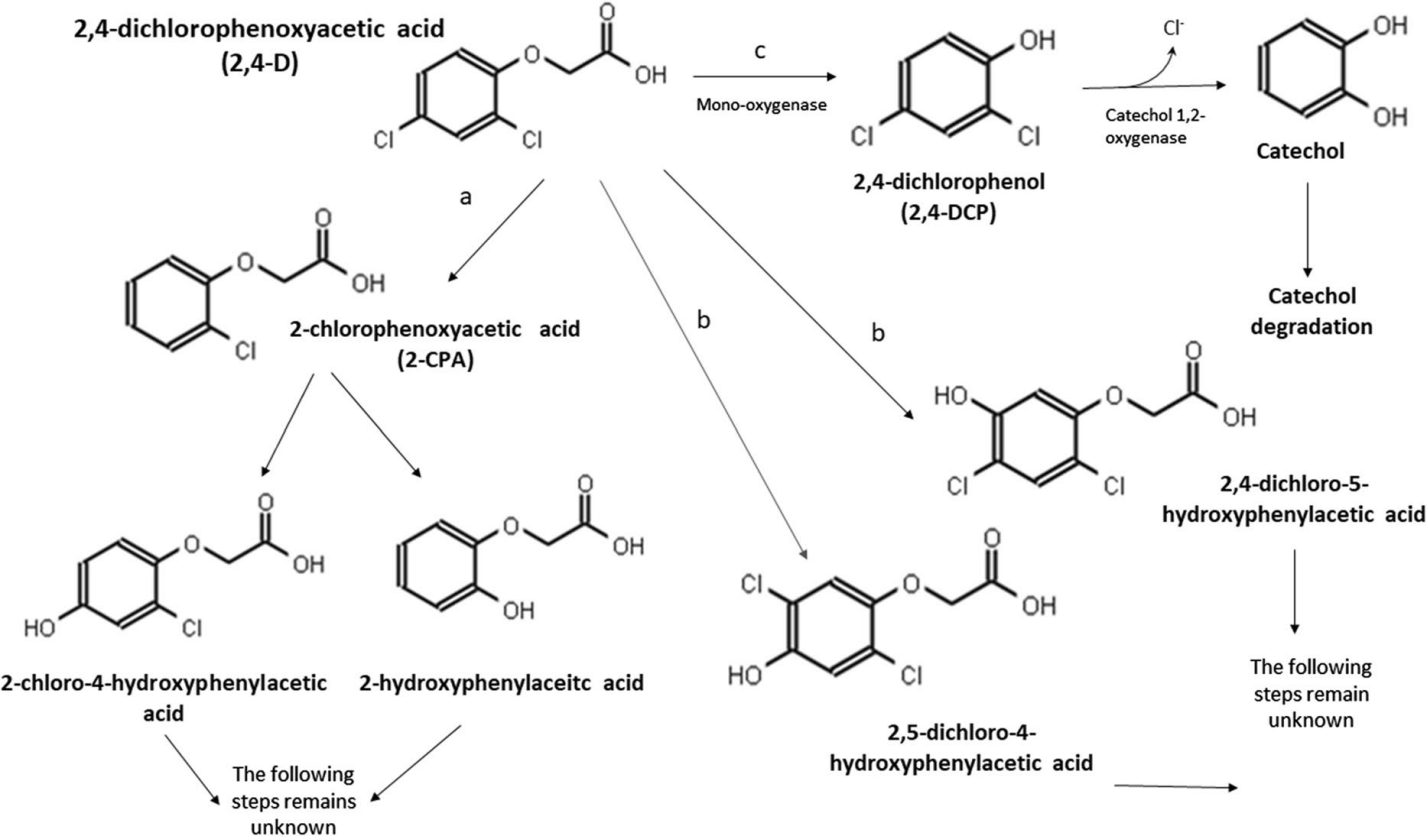
Different pathways for 2,4-D degradation proposed in the literature: **a**. p-chlorophenoxyacetic pathway proposed by Clifford and Woodcock (1964); **b**. pathway proposed by Faulkner and Woodcock (1965) and **c**. 2-DCP pathway proposed by Shailubhai et al. (1983)

Bacterial degradation of 2,4-D has been well characterized. As shown in the EAWAG Biocatalysts/Biodegradation database, only eight bacterial species were sufficient to characterize the different degradation pathways (Gao et al. 2010). However, further insight is needed into the degradation of 2,4-D by fungi. Although species belonging to the divisions Basidiomycota, Ascomycota and Mucoromycota are well-known for their ability to degrade a great variety of xenobiotics, there is little information about the enzymatic pathways involved in the bioconversion process (Serbent et al. 2019). Few authors have so far attempted to describe the main mechanisms underlying fungal degradation or removal of 2,4-D from the environment (Faulkner and Woodcock 1965; Shailubhai et al. 1983; Vroumsia et al. 2005; Ferreira-Guedes et al. 2012; Bhosle and Thore 2016). *Aspergillus niger* (Mulder strain, C.M.I. 31283) was the first fungus reported to degrade the herbicide through the p-chlorophenoxyacetic acid pathway, which has a 2-chlorophenoxyacetic acid (2-CPA) as the main metabolite. This is followed by a hydroxylation reaction that produces 2-chloro-4-hydroxyphenilacetic acid and 2-hydroxyphenylacetic acid (Fig. 4, line a) (Clifford and Woodcock 1946). Two decades later, Faulkner and Woodcock (1965) found that another *A. niger* strain*,* obtained from the Commonwealth Mycological Institute, was also able to degrade 2,4-D. This strain metabolizes the herbicide mainly by hydroxylating its aromatic ring. The main intermediate metabolites are 2,4-dichloro-5-hydroxyphenylacetic acid and 2,5-dichloro-4-hydroxyphenylacetic acid (Fig. 4, line b). According to Shailubhai et al. (1983), the 2,4-dichlorophenol (2,4-DCP) pathway is also used by *A. niger* to degrade 2,4-D. In this case, the main intermediate metabolite is 2,4-DCP, which is then de-halogenated and hydroxylated to catechol (Fig. 4, line c) (Shailubhai et al. 1983). A little earlier, two strains isolated from agricultural soil in France and identified as *Fusarium oxysporum* and *Penicillium rugulosum* had also been reported to have the ability to degrade 2,4-D (Fournier and Catroux 1980), with catechol 1,2-oxygenase likely involved. Since this degradation pathway generates metabolites with similar toxicity to the parent molecule, interaction with other microorganisms is necessary to completely degrade the herbicide. Vroumsia et al. (2005) studied the ability of 90 fungal strains to degrade 2,4-D and 2,4-DCP. Most of them were isolated from soil and decayed wood in France. *Aspergillus penicilloides* and *Umbelopsis isabellina* performed best at degrading 2,4-D, while *Chrysosporium pannorum* and *Mucor generensis* were the most efficient degraders of 2,4-DCP. The latter may be due to the presence of a free hydroxyl group at the C1 position in the aromatic ring of 2,4-DCP.

The fact that some fungi can degrade certain compounds but not their intermediate metabolites, or the reverse, might mean that the processes are catalyzed by different enzymes (Vroumsia et al. 2005). This demonstrates the importance of mixed cultures or microbial consortia, in which microbial groups form a community by establishing cooperative, mutually beneficial relationships (Chaudhary et al. 2018; Pandey et al. 2018) that can promote the complete degradation of toxic metabolites. The varied metabolic abilities displayed by filamentous fungi make them good candidates for the creation of such consortia (Olicón-Hernández et al. 2017).

More recently, Nykiel-Szymańska et al. (2018a) observed that *Umbelopsis isabellina* degrades 2,4-D mainly through the cytochrome P450 pathway. They suggested, moreover, that the main enzymes involved in the degradation of organochlorine herbicides are dehydrochlorination and hydrolytic enzymes, as well as dehydrogenases. This study is the only one so far to elucidate the role of different enzymes in the degradation of 2,4-D.

Finally, Basidiomycota are a large group of macro- and microfungi that play an important role in the carbon and nitrogen cycles, and in the balance of ecosystems (Gadd 2001). They participate in the biodegradation of xenobiotics through extracellular and intracellular mechanisms capable of interacting with and degrading different substrates. The main mechanism is the secretion of oxidative extracellular enzymes such as lacasse, lignin peroxidase (LnP), and manganese peroxidase (MnP). When Basidiomycota sense the presence of xenobiotics in the environment, they synthetize these enzymes, which modify and break down the 2,4-D parent molecules and thus facilitate their adsorption and entry into the fungal cell (Money 2016).

## Background and current situation of 2,4-D research in Argentina

The soils in the Pampa region in Argentina are rich in humic substances, and therefore particularly suitable for the cultivation of several crops. Agrochemicals are an essential element of the agricultural model here (González et al. 2012), and 2,4-D is one of the most used herbicides to control broadleaf weeds. It is applied in formulations such as inorganic or amine salts or as esters, and marketed in the form of emulsions or soluble concentrates. Its use has steadily increased in the country since 1990, except for the period between 2010 and 2012, when it went down by almost 50%. However, by the next season (2012/2013) it had significantly increased again due to the spread of glyphosate-resistant weeds (Caramutti Godoy et al. 2019). Since 2014 its consumption remains stable at 20 million tons, but it is estimated to grow between 10 and 20% in the next few years (CIAFA 2019). Since the ester and isobutyl formulations are highly volatile and disperse rapidly in the environment, their application was banned in 2017 in many Argentinian provinces such as Santa Fe, Chaco, Santiago del Estero, Tucumán, Entre Ríos, and Córdoba (SENASA 2019). The importation and production of these formulations were respectively banned in 2019 and 2020. In 2021, the National Food Safety and Quality Service (SENASA, for its name in Spanish) issued resolutions 466/19 and 875/19, which totally prohibit the commercialization, production, use, and importation of 2,4-D in the whole country (SENASA 2019). Nevertheless, the formulation based on 2,4-D dimetil-amine salt continues to be used.

Several studies carried out in Argentina described the toxicity of 2,4-D in different living beings (Aronzon et al. 2011; Soloneski et al. 2016; Ruiz de Arcaute et al. 2016; Curi et al. 2019). Soloneski et al. (2007) determined the genotoxic effects of different concentrations (10, 25, 50 and 100 mg mL^−1^) in plasma leukocyte cultures and in whole blood from six healthy men. They did so by measuring sister chromatid exchange, cell-cycle progression, and the mitotic index. An increase in the frequency of sister chromatid exchange was observed when using 10 and 50 mg 2,4-D mL^−1^. Cell proliferation suffered a delay in whole blood after treatments with 25 and 50 mg mL^−1^, and mitotic activity was progressively reduced in a dose-dependent manner both in whole blood and the plasma leukocyte cultures. Aronzon et al. (2011) evaluated the toxicity of 2,4-D butyl ester in *Rhinella arenarum* embryos at different developmental stages. The herbicide was teratogenic, and its adverse effects included reduced body size, delayed development, microcephaly, gill agenesis, and abnormal cellular proliferation in the earliest embryonic stage. Ruiz de Arcaute et al. (2016) investigated acute toxicity and genotoxicity caused by 2,4-D in *Cnesterodon decemmaculatus*, a fish species. Concentrations between 252 and 276 mg L^−1^ produced behavioral alterations such as slow motion, slow reaction**,** and abnormal swimming. Moreover, micronuclei were induced, and primary DNA strands were broken. More recently, Curi et al. (2019) surmised the chronic toxicity of exposure to higher concentrations of 2,4-D (between 350 and 2400 mg L^−1^) in *Physalaemus albonotatus* tadpoles. They described oral disc malformations, intestinal abnormalities, histological alterations in the liver structure (e.g., hepatocyte vacuolization, sinusoid enlargement, blood vessel dilation), and a significant increase in the number of melanomacrophages.

After being applied, 2,4-D remains in the soil and is then dispersed in the air and water. Its presence has been reported in different environmental matrices in Argentina. Corcoran et al. (2020) identified it in the La Brava Lake (Buenos Aires), the Suquia River, and the Calamuchita River (Córdoba), at concentrations lower than 320 ng L^−1^. Peluso et al. (2020) informed concentrations between 1 and 4 μg L^−1^ in the Paraná River (Buenos Aires). To protect aquatic wildlife, national regulations allow no more than 4 μg L^−1^ in surface waters (Act 24051/92 on Hazardous Waste (WHO, 1993)). Other Argentinian reports have focused on bacterial degradation of 2,4-D in different environments, like the southern Pampa region (Buenos Aires) (Zabaloy and Gómez 2008; Zabaloy et al. 2010), the Sauce Grande River (Buenos Aires) (Zabaloy and Gómez 2013), and the town of Colón, also in the Humid Pampa region (Buenos Aires) (Merini et al. 2007). In 2012, a new *Delftia* strain isolated from a river in Buenos Aires was able to degrade 2,4-D in vitro (González et al. 2012). Nevertheless, nothing is known about the genes and enzymes involved. Moreover, despite the biotechnological potential of fungi, no 2,4-D-degrading fungal strains have been isolated in Argentina. There is a great gap to be filled in this respect, and the same can be said for what we know about the genes and/or enzymes potentially involved in the fungal pathways of 2,4-D degradation.

## Selection of microorganisms from contaminated sites with potential for application in bioaugmentation strategies

The microbial communities that inhabit sites contaminated with chlorinated herbicides have evolved to tolerate and even thrive under high concentrations of 2,4-D. They may therefore be able to remove the herbicide from the environment by using it as a nutrient source (Kumar et al. 2021).

Nevertheless, when a certain environment has only recently been polluted with 2,4-D, the native microbial community is not yet adapted, and its members are less likely to remove the herbicide. A good way to enhance removal in these cases is to introduce fungal strains capable of tolerating and removing 2,4-D. These strains may naturally contain the necessary genes or may have been genetically modified (GMMs) to contain them. To implement a biotechnological strategy of this kind, the first step is to isolate native fungal strains with the ability to tolerate 2,4-D (Bhadouria et al. 2020). Isolation begins when samples from contaminated sites are enriched to encourage the growth of fungi that can degrade 2,4-D. To achieve this, the liquid growth medium is incubated under conditions which favor the development of fungi with the ability to use the herbicide as a source of energy and carbon. The fungi that grew during the enrichment phase are isolated on solid media with a similar formulation to that of the enriched liquid medium. Then, they are identified and characterized, and a selection is made of those which can tolerate not only environmental levels of 2,4-D, but even higher concentrations (as in the aftermath of a pesticide spill). In the laboratory, they are tested for their ability to use the herbicide as a source of nutrients, in a culture medium or a natural substrate containing 2,4-D as a carbon source. The assays are subsequently scaled up to simulate actual environmental conditions. These in situ assays can reliably demonstrate whether the isolates are suitable to be applied as biodegradation agents. Once the most apt strains have been selected, further studies determine the best application conditions as part of a bioaugmentation strategy. Bioaugmentation, which is widely used in bioremediation, consists of adding exogenous microorganisms to a site that needs to be remediated, with the aim of improving the contaminant removal rate efficiency (Kumar et al. 2021). The success of bioaugmentation depends on several factors. After microorganisms with sufficient biotechnological potential have been found, their likelihood of competing against native microbiota should be explored. Following a period of acclimatization, the incorporated fungal strains should ideally interact with the native microbiota, so that mechanisms such as biosorption, bioabsorption, biotransformation, and/or degradation may take place and lead to the removal or neutralization of the herbicide. Whether these processes occur separately or simultaneously will depend on the metabolic capacity of the strains that make up the microbial community (Bokade et al. 2021).

## Argentinian regulations for the introduction of microorganisms into the environment

The introduction of microorganisms into the environment can be controversial due to the ecological disturbances it may cause. For this reason, this practice is regulated by different legal frameworks. Both genetically modified microorganisms (GMMs) and bioinputs may be introduced (Burachik and Traynor 2002; Flint et al. 2000). According to the Ministry of Agriculture, Livestock and Fisheries in Argentina (MAGyP), bioinputs are products made from microorganisms (bacteria, fungi, and viruses), macroorganisms (beneficial insects), plant extracts, and/or compounds derived from natural or biological sources, which are applied in agriculture, the agro-food sector, agricultural industries, the production of agroenergy, and environmental sanitation. The concept includes biofertilizers, biocontrollers, phytostimulants, bioremediators, biotransformers for the treatment of agricultural subproducts, and bioinputs to produce bioenergy. These products are eco-friendly and improve agro-industrial productivity (MAGyP 2022a).

The Argentinian regulatory framework for biotechnological products is one of the oldest systems of its kind in the world. The National Commission on Agricultural Biotechnology (CONABIA) was created in 1991, under resolution 124/91 from the MAGyP. Another resolution (763/11) regulates the implementation of general laws related to genetically modified organisms (GMOs) (CONABIA 2022; MAGyP 2022a). The CONABIA is made up of members from both the public and private agricultural sectors. It is an inter-institutional and multidisciplinary organization, whose main function is to offer technical support for the design and management of biosafety protocols aimed at introducing and releasing transgenic material into the environment. In other words, this committee seeks to guarantee that the GMOs incorporated into the environment are safe for the country’s agricultural ecosystems and for human and animal health (MAGyP 2022b).

On the other hand, the Advisory Committee on Bioinputs for Agricultural Use (CABUA) is an entity that regulates the introduction of bioinputs into the environment. It was created under the supervision of CONABIA through resolution 7/2013 from the Secretariat of Agriculture, Livestock and Fisheries (SAGyP). Resolution 41/2021 placed it within the Department of Coordination of Innovation and Biotechnology belonging to the National Directorate of Bioeconomy, and increased its roles and actors. It provides technical support to ensure the quality, efficacy, and biosafety of agricultural bioinputs, and it aims to create an adequate legal framework for their use, management, and disposal. Table 3 summarizes the specific regulations for biotechnological products established by the MAGyP (CABUA 2021; MAGyP 2022a).

**Table 3:** Main specific regulations on biotechnological products of Argentina

Biotechnological products	Regulatory framework and descriptions
GMOs	Resolution 763/2011: general aspects of activities involving GMOs
	Resolution 112/2016: appointment of main members of CONABIA and their respective functions in the committee
GMMs	Resolution 5/2018: authorization of experimental activities involving living or dead GMMs for agro-industrial applications
	Resolution 52/2019: procedures for the evaluation of living or dead GMMs released into the environment for agroindustrial applications
Agricultural bioinputs	Resolution 7/2013: creation of CABUA under the supervision of CONABIA
	Resolution 41/2021 by the Secretariat of Food, Bioeconomy and Regional development in replacement of resolution 7/2013
	Resolution 105/2019: action plan for agricultural bioinputs

*GMOs* genetically modified organisms, *GMMs* genetically modified microorganisms, *CONABIA* National commission on agricultural biotechnology, *CABUA* advisory committee on bioinputs for agricultural use. *Source* MAGyP 2022a, b

In general, CONABIA and CABUA set certain requirements for the registration of GMMs and bioinputs in our country, which include the identification and classification of the microorganism. This means information must be provided on the kind of biologic agent it is (fungus, bacterium, virus), its metabolic characteristics, its ecological distribution, and its genetic stability (Desai et al. 2016; Ravensberg 2011). The most important part of the registration process, however, is the toxicological evaluation. These are studies that help to predict the pathogenicity, toxicity, and infectivity of GMMs and bioinputs once they are applied in the environment (Desai et al. 2016). To respect the current situation of 2,4-D research in Argentina, Carles et al. (2021) studied bacterial strain *Cupriavidus necator* JMP134 for its ability to bioremediate the herbicide in soil microcosms. The strain accelerated the mineralization of 2,4-D in situ, and its introduction did not significantly affect the biodiversity of the native bacterial community. To date, this is the only study carried out in Argentina on the potential application of a microorganism to bioremediate 2,4-D. Despite the encouraging results obtained, the strain has not been made commercially available.

## Future perspectives on the use of 2,4-D in Argentina

The use of 2,4-D is expected to increase in Argentina in the next few years, due to the constant demand for better quality, nutrient-rich food, and the expansion of agricultural borders (Principiano and Acciarresi, 2018). However, the existing controversy about its carcinogenicity means that more provinces are planning to restrict its production and use. Although 2,4-D does fulfill an important role in the current agricultural model, more should be known about its potential degradation by filamentous fungi isolated in our country. This would make it possible to design remediation strategies aimed at offsetting the herbicide’s negative impact.

## Conclusion

Fungi have been extensively studied in terms of their capacity to degrade and detoxify xenobiotic compounds. They are known to deploy different mechanisms to remove herbicides, such as bioabsorption, biosorption, biotransformation, and/or biodegradation. Their efficient enzymatic machinery plays a crucial role in biodegradation, because it can break down parent molecules and make them less toxic for the environment and for human and animal health. However, the ability of fungi to degrade 2,4-D has not been as explored as that of bacteria, and little is known about the metabolic and degradation pathways through which these enzymes achieve their task. The presence of 2,4-D in the environment makes it a subject of scientific interest. Fungi have important biotechnological potential for its bioremediation. For that to happen, new fungal strains need to be isolated and studied for their ability to bioremediate contaminated environments, within the current legal frameworks. A better understanding of the enzymes, pathways, and mechanisms behind this ability is also paramount for the design of effective strategies aimed at detoxifying natural matrices such as soil, water, or wastewaters.
